# Fast, Simple and Accurate Method for Simultaneous Determination of α-Lipoic Acid and Selected Thiols in Human Saliva by Capillary Electrophoresis with UV Detection and pH-Mediated Sample Stacking

**DOI:** 10.3390/molecules30153129

**Published:** 2025-07-25

**Authors:** Urszula Sudomir, Justyna Piechocka, Rafał Głowacki, Paweł Kubalczyk

**Affiliations:** 1 Department of Environmental Chemistry, Faculty of Chemistry, University of Lodz, 163/165 Pomorska Str., 90-236 Łódź, Poland; urszula.sudomir@edu.uni.lodz.pl (U.S.); rafal.glowacki@chemia.uni.lodz.pl (R.G.); 2 Doctoral School of Exact and Natural Sciences, University of Lodz, 12/16 Banacha Str., 90-237 Łódź, Poland

**Keywords:** capillary electrophoresis, 2-chloro-1-methyllepidinium tetrafluoroborate, lipoic acid, pH-mediated sample stacking, saliva, aminothiols

## Abstract

This report presents the first method for simultaneous determination of the 2-*S*-lepidinium derivatives of total α-lipoic acid (LA), homocysteine (Hcy), cysteinylglycine (CysGly), and cysteine (Cys) in human saliva, using capillary electrophoresis with pH-mediated sample stacking and ultraviolet detection (CE-UV) at 355 nm. Electrophoretic separation is carried out at 20 kV and 25 °C using a standard fused silica capillary (effective length 91.5 cm, inner diameter 75 µm). The background electrolyte consists of 0.5 mol/L lithium acetate buffer, adjusted to pH 3.5 with 0.5 mol/L acetic acid. The limit of quantification was determined to be 1 µmol/L for LA and 0.17 µmol/L for Hcy, 0.11 µmol/L for CysGly, and 0.10 µmol/L for Cys in saliva samples. Calibration curves demonstrated linearity over the concentration range of 3 to 30 µmol/L for all analytes. Method precision did not exceed 4.7%, and accuracy ranged from 87.9% to 114.0%. The developed method was successfully applied to saliva samples from eleven apparently healthy volunteers to determine the content of LA, Hcy, CysGly, and Cys. The Hcy, CysGly, and Cys concentrations ranged from 0.55 to 13.76 µmol/L, 0.89 to 9.29 µmol/L, and 1.73 to 12.99 µmol/L, respectively. No LA-derived peaks were detected in the native saliva samples.

## 1. Introduction

**Figure 1:**
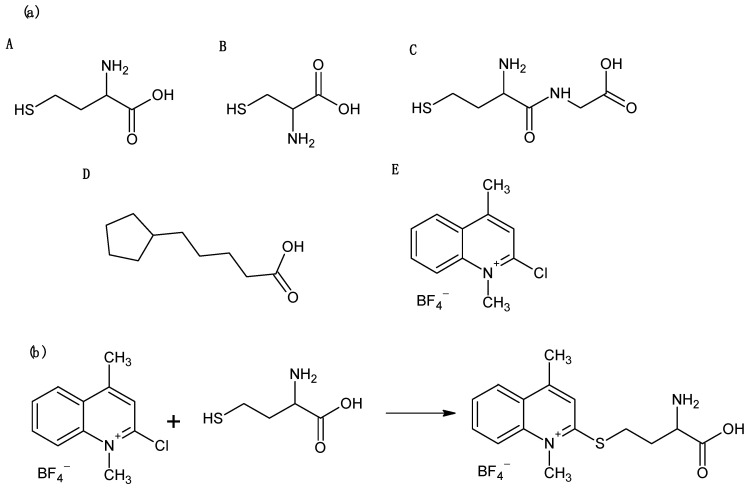
(**a**) Structures of (**A**) homocysteine, (**B**) cysteine, (**C**) cysteinylglycine, (**D**) lipoic acid, and (**E**) 2-chloro-1-methyllepidinium tetrafluoroborate (CMLT) (**b**) Schematic representation of the reaction involved in the chemical derivatization of Hcy with CMLT, yielding 2-*S*-lepidinium derivative of Hcy.

Homocysteine (Hcy), cysteine (Cys), cysteinylglycine (CysGly), and lipoic acid (LA) [1,2,3] (Figure 1a), classified as low molecular weight sulfur compounds, play a key role in maintaining redox balance in the human body. These compounds exhibit antioxidant properties, protecting cells from oxidative stress, which is associated with the development of various lifestyle-related diseases, such as diabetes, cancer, and neurodegenerative disorders (e.g., Alzheimer’s and Parkinson’s disease). Monitoring the concentrations of Hcy, Cys, CysGly, and LA in biological fluids has significant diagnostic value for various disorders in the human body, as these compounds can serve as markers of oxidative stress, treatment response, and disturbances of sulfur compound metabolism. In order to determine the total content of low-molecular-weight sulfur compounds in saliva, a three-stage sample preparation protocol is recommended. The first step involves the reduction of disulfide bonds, resulting in the release of sulfhydryl groups (-SH), which are then available for further chemical reactions. The second step is derivatization, a chemical modification of analytes involving the formation of stable derivatives and the blocking of reactive thiol groups, thereby improving the selectivity and sensitivity of the assays. The final step is the removal of proteins and other salivary components that may interfere with the analytical signal, thus enabling accurate detection of the target compounds. In recent years, saliva has gained increasing attention as a matrix for clinical research. This interest stems from several advantages it offers over traditionally used biological fluids such as urine and serum [4]. Saliva reflects the current physiological state of the patient and contains a wide spectrum of compounds whose presence and concentration are often correlated with pathological conditions and with serum levels [5].

So far, a number of methods using capillary electrophoresis (CE) with different types of detection have been developed for the determination of sulfur compounds in biological samples [6]. Previous CE methods for the quantitative determination of these compounds have been mainly based on the analysis of blood [7,8,9] and urine [10]. However, saliva analysis for Hcy, Cys, LA, and CysGly was performed using other techniques, such as high-performance liquid chromatography (HPLC) with ultraviolet (UV) detection, gas chromatography (GC) with mass spectrometry (MS), and ionic liquid chromatography (IP-HPLC) with UV detection [11,12,13]. Just one attempt to determine total glutathione (GSH) in saliva, which belongs to the group of low-molecular-weight sulfur compounds, using CE with laser-induced fluorescence (LIF) detection has also been described [14]. However, in the method developed by Hodáková et al., the detection of GSH in saliva was only possible after the sample was spiked with a GSH standard. To date, however, no method has been published for simultaneous determination of the total content of Hcy, Cys, LA, and CysGly in human saliva using CE with UV detection. Therefore, it is necessary to fill the research gap by developing an effective, sensitive, and environmentally friendly analytical method. The main objective of this work was to develop a method using CE-UV for the determination of a wide range of low-molecular-weight sulfur compounds—such as LA, Hcy, Cys, and CysGly in human saliva. The intermediate objectives included optimizing the sample preparation steps, fully validating the method, applying it to real samples, and assessing the method’s environmental greenness.

## 2. Results and Discussion

The paper presents a CE-UV-based method for the simultaneous determination of total Hcy, Cys, CysGly, and LA in human saliva. During the design of the method, a strong emphasis was placed on greening the analytical procedures, taking into account each of the twelve principles of Green Analytical Chemistry (GAC) [15]. The following (sub)sections of the report provide all relevant information related to the development, validation, and in-study use of the assay described herein.

### 2.1. Sample Preparation

Sample preparation procedure involves simultaneous disulfide bond reduction with tris(2-carboxyethyl)phosphine hydrochloride (TCEP) and pre-column derivatization with 2-chloro-1-methyllepidinium tetrafluoroborate (CMLT), followed by sample deproteinization via centrifugation, preceded by sample acidification with perchloric acid (PCA). In general, these steps are typical of these kinds of methods [2,16,17]. The procedure is partly based on our previously published HPLC-UV assay by Stachniuk et al. [11], which enables the determination of Hcy, Cys, GSH, CysGly, γ-glutamyl-cysteine (γ-Glu-Cys), and N-acetyl-cysteine (NAC) in saliva, urine, and plasma samples. Optimization of the sample preparation conditions was investigated using the HPLC-UV assay published by Piechocka et al. [18].

#### 2.1.1. Preliminary Considerations

The normal saliva pH is 6.0–7.0 and can vary depending on various factors [2,19]. Since Stachniuk et al. [11] recognized that the yield of the derivatization reaction of Hcy, Cys, GSH, CysGly, γ-Glu-Cys, and NAC with CMLT is pH-dependent, adjusting the pH of the sample is necessary to minimize its contribution to the recovery losses in this method. As reported previously [11], phosphate buffer (PB) 0.2 mol/L and pH 7.8 was chosen to adjust the sample pH. It was established that a minimum ratio of 1:2 (saliva to buffer, *v*/*v*) is necessary to achieve a pH in the mixture equivalent to that of the buffer, regardless of the source or the initial pH of the saliva sample, which can range from 5.5 to 8.0. As described in the following sections, the most satisfactory results were obtained when 50 µL of saliva was mixed with 100 µL of 0.2 mol/L PB buffer (pH 7.8).

#### 2.1.2. Disulfide Bond Reduction

**Figure 2:**
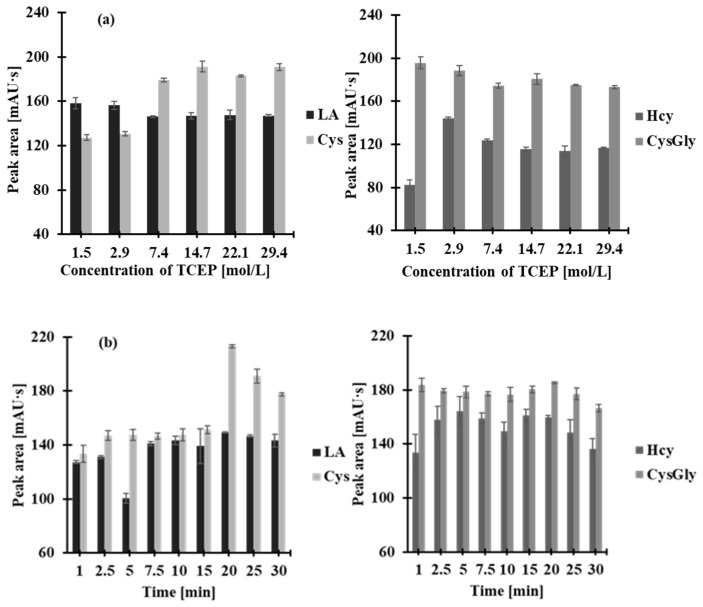
The effect of (**a**) TCEP concentration in the reaction mixture and (**b**) reaction time, on peak area of a 2-*S*-lepidinium derivative of Hcy, Cys, CysGly, and LA. Error bars represent the standard deviation (*n* = 3).

In order to assess the total content of Hcy, Cys, CysGly, and LA in saliva, samples must first be treated with a disulfide reducing agent to prevent losses of the analytes. In this study, TCEP was selected to cleave disulfide bonds, as recommended in the literature [2,11], since the phosphine effectively reduces all oxidized thiols and separates them from salivary proteins at room temperature [11,12]. The generation of free thiols from disulfides was also important for making them accessible to the derivatization agent. The main factors affecting the disulfide reduction yield, namely, the quantity of TCEP and reaction time, were optimized. Their impact on the reaction was tested with TCEP concentrations ranging from 1.47 to 29.41 mmol/L saliva samples and reaction times ranging from 0 to 30 min. It was observed that the peak area corresponding to the particular analyte increased progressively in parallel with the rise in TCEP concentration from 1.47 to 7.35 mmol/L. Beyond this concentration, there were generally no significant changes, except for the CysGly-delivered signal (Figure 2a). For routine analysis, 50 µL of saliva was treated with 5 µL 0.25 mol/L TCEP, resulting in a final concentration of 7.35 mmol/L in the saliva samples. A larger excess of TCEP was excluded as it contributed to additional baseline noise. Under these conditions, the reaction was completed in 2 min at room temperature, almost immediately after mixing the reagents (Figure 2b).

#### 2.1.3. Derivatization

**Figure 3:**
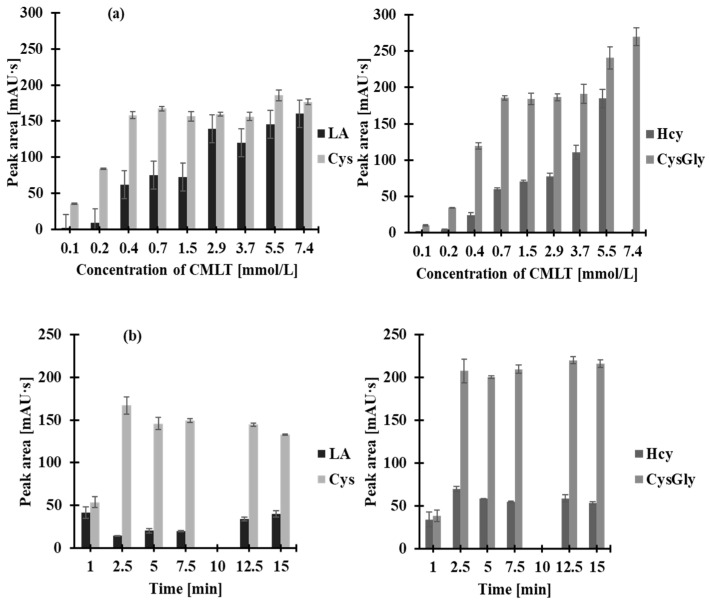
The effect of (**a**) CMLT concentration in the reaction mixture and (**b**) reaction time, on the peak area of the 2-*S*-lepidinium derivative of Hcy, Cys, CysGly, and LA. Error bars represent the standard deviation of the data (*n* = 3).

The presented CE-UV assay is based on the derivatization of analytes with the thiol-specific reagent CMLT, which contains an active halogen atom as its reactive moiety (Figure 1b). Derivatization with CMLT is particularly important for enhancing UV detectability and improving chemical stability, and also for introducing a permanent positive charge on the nitrogen atom in the lepidinium moiety, thereby ensuring consistent electrophoretic mobility across a broad pH range. Thus far, the effectiveness of CMLT as a derivatizing agent has been demonstrated in the determination of 2-mercaptoethanesulfonate [20], Nε-homocysteinyl-lysine [21], γ-Glu-Cys [11,21], Hcy, Cys, GSH, CysGly, and NAC [11] in human plasma [11,20,21], urine [11], and saliva [11] by CE-UV [20] and HPLC-UV [11,21]. It has been shown that CMLT reacts almost instantaneously (<3 min) at room temperature, with the low-molecular-weight sulfur-containing compounds mentioned above in 0.2 mol/L PB buffer (pH 7.4–7.8) yielding the corresponding UV-absorbing 2-*S*-lepidinium derivatives. In the present study, the effects of CMLT quantity in the reaction mixture and reaction time on derivatization efficiency were studied, as no data on this topic were available for LA. The reaction course was examined at room temperature over a range of 0–15 min, using CMLT solutions at concentrations varied from 2.5 to 250 mmol/L, which provided CMLT concentrations in saliva samples from 0.07 to 7.35 mmol/L. The best results were obtained when 5 µL of 0.025 mol/L CMLT was added, resulting in a final concentration of 0.74 mmol/L for the saliva samples (Figure 3a). Under these conditions, processing the samples for 2 min at ambient temperature was sufficient to effectively derivatize all reduced forms of the analytes with high precision (Figure 3b). To the best of our knowledge, this is the first report of such data regarding LA.

#### 2.1.4. Integration of Disulfide Bond Reduction with Derivatization

**Figure 4:**
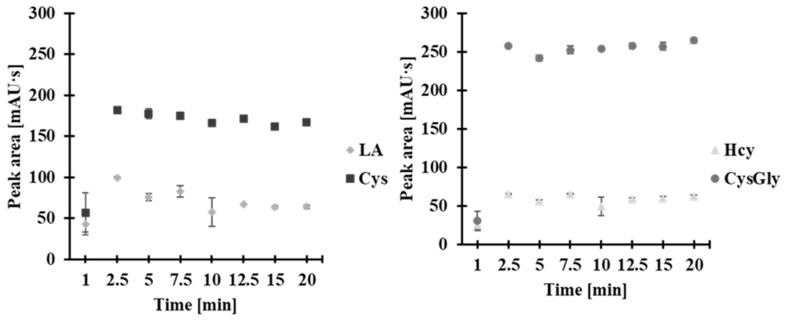
The effect of time on the peak area of the 2-*S*-lepidinium derivative of Hcy, Cys, CysGly, and LA upon simultaneous disulfide bond reduction and derivatization. Error bars represent the standard deviation of the data (*n* = 3).

As the fourth principle of GAC states, the number of analytical steps should be minimized to the greatest extent possible, resulting in savings of material, energy, and time, among other benefits [22]. Accordingly, significant efforts have been made toward the integration of analytical steps. In the present study, the reduction of disulfide bonds was carried out simultaneously with derivatization. Samples were treated with the mixture containing 0.25 mol/L TCEP and 0.025 mol/L CMLT. The time course of the process was examined in the range of 0–15 min at room temperature. It was observed that the signal from the 2-*S*-lepidinium derivatives of Hcy, Cys, CysGly, and LA reaches its maximum just 2 min after mixing the reagents and remains stable thereafter (Figure 4). For routine analysis, disulfide bond reduction and derivatization were conducted simultaneously, as this approach was found to be superior in terms of workflow simplification and improvement of batch-to-batch reproducibility. Additionally, a 5 min processing time was chosen considering the timeframe required for processing a batch of 24 samples. These results are consistent with the literature and support the idea that highly reproducible results and good reaction yields for LA, lipoyllysine, Cys, GSH, CysGly, and Hcy can be obtained when samples are simultaneously subjected to disulfide bond reduction with TCEP and derivatization with 1-benzyl-2-chloropyridinium bromide, a compound with an activated halide similar to CMLT [17].

**Figure 5:**
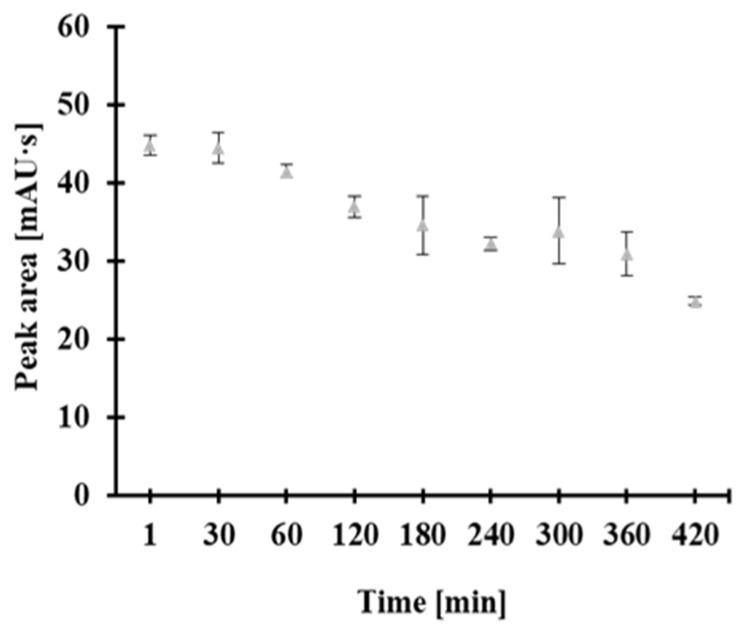
Stability of a water solution containing TCEP (0.25 mol/L) and CMLT (0.025 mol/L) at room temperature as a function of time, expressed as a peak area of 2-*S*-lepidinium derivative of Hcy. Error bars refer to the standard deviation of the data (*n* = 3).

During the study, the utility of the mixture containing 0.25 mol/L TCEP and 0.025 mol/L CMLT was also tested. The stability of the reagent was evaluated at 4 °C across one working day. To determine the maximum time, a processed sample could be treated with the mixture of TCEP and CMLT without noticeable changes in analyte content. Study samples were prepared at preselected time intervals using the same reagent. It was found that the efficiency of the combined disulfide bond reduction and derivatization process decreased when samples were treated with a 60 min old mixture of TCEP and CMLT, as evidenced by a decrease in the peak area of the 2-*S*-lepidnium derivative of Hcy (Figure 5). Based on these results, it was concluded that the reagent should be prepared fresh and used without delay.

#### 2.1.5. Removal of Proteins

Human saliva consists primarily of 99.5% water, while the rest is proteins, among others [2,19]. In order to reduce the complexity of test samples and limit the possibility of clogging the CE capillary, the assay involves sample deproteinization. Based on our earlier findings [2,11], salivary proteins were separated from the other sample components by means of acidification with 3 mol/L PCA, followed by centrifugation at reduced temperature to obtain clear supernatant, and such an approach was beneficial for workflow simplification. Sample acidification was also essential to terminate the derivatization reaction and stabilize the obtained products [11]. Since efficient protein precipitation and sample acidification were achieved by mixing the saliva sample with 3 mol/L PCA and minimal crashing at a 5:1 volume ratio, no further work was undertaken to optimize the deproteinization step.

#### 2.1.6. Stability of Processed Samples

The total time and conditions under which processed samples are stored until completion of analysis were evaluated. The stability of the 2-*S*-lepidinium derivatives in the given matrix was assessed in a temperature-controlled autosampler set to 25 °C, 4 °C, −20 °C, and −80 °C. It was found that their content remains stable for at least one month under all examined conditions, regardless of the concentration of the particular analyte within the quantitation range. The observed drops in concentration were 86.2%, 87.3%, 84.1%, and 87.1% for LA, Hcy, CysGly, and Cys, respectively (Figure S1a–d). A freeze–thaw stability test also showed that reliable and valid CE-UV data could be obtained even after five thawing cycles (Figure S2). These results are consistent with the literature data and support the idea that 2-*S*-lepidinium derivatives of Hcy, Cys, and CysGly are highly stable compounds [11,21]. Regarding the 2-*S*-lepidinium derivative of LA, this is the first report on this topic, to the best of our knowledge. In this way, sample handling and management effort could be significantly reduced due to the excellent stability of the analytes under experimental conditions. The stability of 2-*S*-lepidinium derivatives allows for the preparation of large batches of samples without the need for rapid processing through the CE-UV method.

Overall, our research led us to the conclusion that CMLT is a suitable derivatizing agent not only for low-molecular-weight aminothiols [11,20,21] but also for LA. An analytically advantageous feature is the excellent stability of each analyte-delivered CMLT derivative under experimental conditions, which allows for preparation of large batches of samples, minimizing effort. Moreover, the attractiveness of the assay presented herein relies on a straightforward sample preparation procedure and relatively short process time (~13 min) (Table 1). In this regard, our method is more convenient than other available HPLC-based assays, where the process takes more than 21 min [2]. In addition, the new procedure involves fewer steps than other methods. Particularly, the integration of disulfide bond reduction with the derivatization process has proven beneficial, as it reduces labor intensity and maximizes sample throughput. Moreover, the ability to perform chemical analysis on a very small scale, combined with the relatively low consumption of hazardous chemicals and laboratory plastic disposables, represents an additional advantage. The process requires only 120 μL of inexpensive, though not commercially available, chemicals.

### 2.2. Optimization of Electrophoretic and Detection Conditions

#### 2.2.1. Buffer Selection

Six electrolyte solutions were tested as background electrolyte (BGE): 0.3 mol/L acetate buffer (pH 3.5), 0.3 mol/L phosphate-citrate buffer (pH 3.5), 0.3 mol/L phosphate buffer (pH 3.5), acetate-citrate buffer (pH 3.5), 0.3 mol/L citrate buffer (pH 3.5), and 0.3 mol/L formate buffer (pH 3.5). The best peak parameters (height, area, repeatability, and resolution) were achieved using acetate buffer. Then, the concentration and pH of this buffer were optimized.

#### 2.2.2. Optimization of BGE Concentration and pH

**Figure 6:**
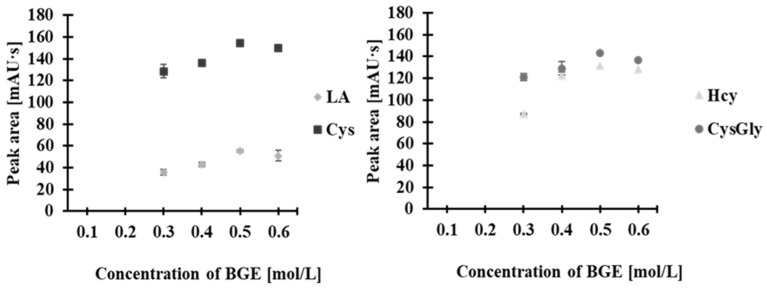
Effect of BGE concentration on peak area of 2-*S*-lepidinium derivatives of Hcy, Cys, CysGly, and LA. Error bars represent standard deviation of data (*n* = 3).

The first parameter optimized was the concentration of the BGE. The effect of BGE concentration on peak height, area, shape, resolution, and repeatability of the analytical signals was examined. BGE concentrations (pH 3.5) were tested in the range of 0.1–0.6 mol/L with increments of 0.1 mol/L. At BGE concentrations between 0.1 and 0.2 mol/L, no analyte signals were observed. The highest peaks (Figure 6) were obtained with an acetate buffer concentration of 0.5 mol/L, which was used for further experiments.

Next, the effect of BGE pH on peak height, repeatability, and resolution was examined. A 0.5 mol/L acetate buffer with pH values varying from 3.0 to 3.8 was tested. Peaks from LA, Hcy, CysGly, and Cys did not separate at pH values above 3.4. Although higher signals were observed at pH values above 3.6, adequate peak separation was not achieved. Therefore, to improve peak separation, a 0.5 mol/L acetate buffer composed of 0.5 mol/L CH_3_COOH and 0.5 mol/L CH_3_COOLi, adjusted to pH 3.5, was selected for further experiments.

#### 2.2.3. Electrophoresis with pH-Mediated Stacking

The most commonly cited limitation of CE with UV detection is its relatively low concentration sensitivity, particularly when compared to HPLC [23]. One effective approach to improve the sensitivity of CE is the use of online sample preconcentration techniques, such as pH-mediated stacking [9,24]. The mechanism behind this preconcentration technique is well-established and relatively easy to implement [23,24], which facilitated its successful integration into our methodology. Given the very low concentrations of the target analytes in saliva, sample preconcentration was performed directly inside the capillary to enable their efficient detection. The previously selected acetate buffer, composed of CH_3_COOH and CH_3_COOLi, proved to be well suited for application in the aforementioned stacking technique.

#### 2.2.4. Optimization of the Sample and Acid Injection Time

The next step was to investigate the effect of the sample volume introduced into the capillary on the height of the analytical signal. To do this, we introduced the sample into the capillary electrokinetically at a constant voltage (20 kV) and constant acid introduction rates (20 kV, 80 s), varying the sample introduction time between 20, 40, 60, 80, 100, 120, and 140 s.

**Figure 7:**
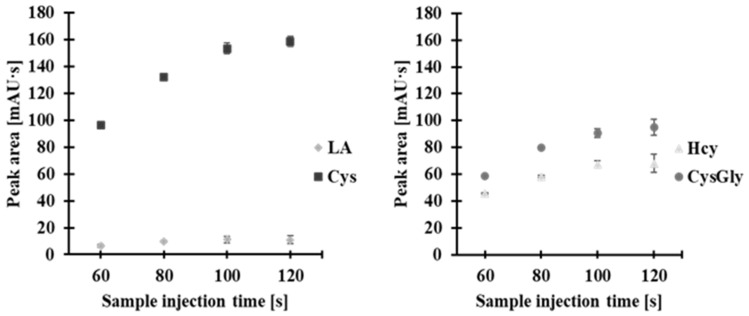
Effect of sample injection time on peak area of 2-*S*-lepidinium derivatives of Hcy, Cys, CysGly, and LA. Error bars represent the standard deviation of the data (*n* = 3).

Sample introduction for 20 and 40 s was probably too short, as we observed peaks with low signals that were not separated from the baseline. On the other hand, sample introduction for 140 s likely caused capillary overload, as the peaks did not separate. As shown in Figure 7, the highest analytical signals with baseline separation were achieved when the sample was introduced for 100 s. Therefore, we used this sample introduction time in further experiments.

**Figure 8:**
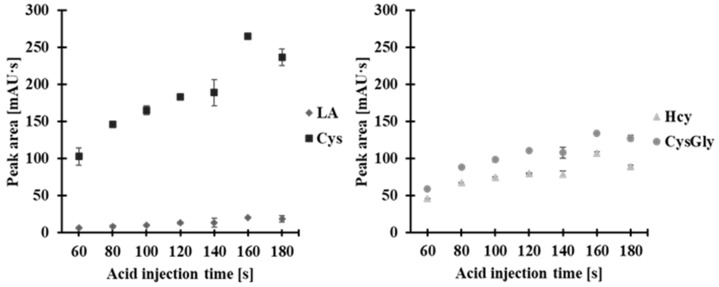
Effect of acid injection time on peak area of 2-*S*-lepidinium derivatives of Hcy, Cys, CysGly, and LA. Error bars represent the standard deviation of the data (*n* = 3).

The effect of the volume of acid introduced into the capillary on peak separation was then investigated. To do this, we introduced the sample into the capillary in an electrokinetic manner, varying the acid introduction time (0.1 mol/L HCl) while keeping the voltage constant at 20 kV and the sample introduction rates constant at 100 s (20 kV). Acid was introduced for varying durations: 40, 60, 80, 100, 120, 140, 160, and 180 s. Introduction times of 40 s and 60 s were probably too short and resulted in peaks with low signal and poor baseline separation. On the other hand, acid introduction times of 140–180 s likely caused capillary overload, as the peaks did not separate. As shown in Figure 8, the highest analytical signals with baseline separation were obtained when the acid was introduced for 100 s. This method was then used during subsequent experiments.

#### 2.2.5. Optimization of Acid Concentration

Once the optimal time of acid introduction into the capillary was determined, the effect of HCl concentration on peak separation and repeatability was examined. These tests were carried out under constant sample and acid injection conditions (20 kV, 100 s for both). The following HCl concentrations were tested: 0.05 mol/L, 0.1 mol/L, 0.15 mol/L, 0.2 mol/L, 0.3 mol/L, 0.4 mol/L, 0.5 mol/L, 0.6 mol/L, 0.7 mol/L, 0.8 mol/L, 0.9 mol/L, and 1.0 mol/L.

**Figure 9:**
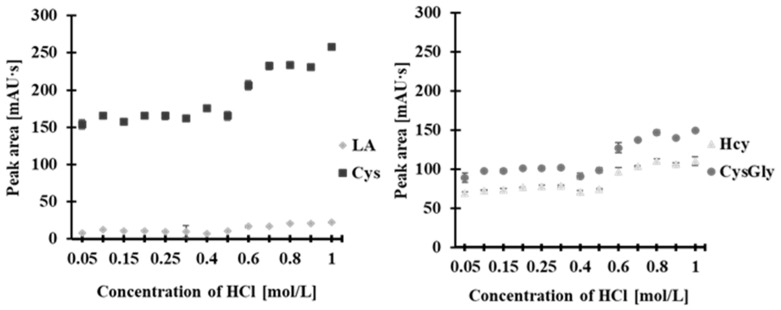
Effect of acid concentration on the peak area of 2-*S*-lepidinium derivatives of Hcy, Cys, CysGly, and LA. Error bars represent the standard deviation of the data (*n* = 3).

As shown in Figure 9, the highest analytical signals were obtained with HCl at a concentration of 0.8 mol/L. When the HCl concentration was below 0.15 mol/L, the peaks for LA, Hcy, Cys, and CysGly did not separate satisfactorily. However, starting from a concentration of 0.25 mol/L, baseline separation was achieved. The best results in terms of satisfactory separation and peak migration time were obtained with HCl concentration of 0.6 mol/L. Therefore, this concentration was selected for further studies.

#### 2.2.6. Sensitivity Enhancement Factor

To determine the concentration of the analytes, a pre-concentration step based on online sample stacking within the CE system was required. A sensitivity enhancement factor (SEF) was used to evaluate the efficiency of the stacking process. The SEF is calculated by comparing the peak height or peak area obtained using a method with a concentration step to that obtained using a procedure without this step. The SEF was calculated using the following equation:

$$SEF=\frac{h^{\prime }}{h}\times \frac{C}{C^{\prime }}$$

where *h*′ is the peak height of the analyte for saliva analysis after the concentration step, *h* is the peak height of the analyte for saliva analysis without the concentration step, *C*′ is the analyte concentration in saliva sample, which was analyzed with the concentration step, and *C* is the analyte concentration in saliva sample, which was analyzed without the concentration step.

**Figure 10:**
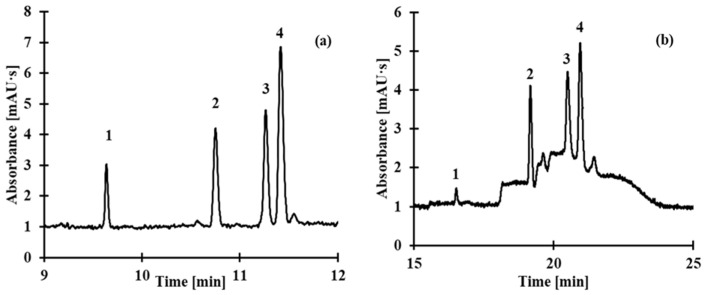
Representative electropherograms of human saliva obtained using the stacking method (**a**) and a simple CE method (sample volume below 2% of the capillary capacity) that does not involve any concentration technique (**b**). The saliva samples were spiked with known amounts of analyte: 3 μmol/L saliva sample for the stacking method (**a**) and 70 μmol/L saliva sample for the simple CE method (**b**). Peaks: 1 LA, 2 Hcy, 3 CysGly, and 4 Cys.

Saliva samples were prepared following the procedure described in the *Saliva Sample Preparation* section, with each sample prepared in five replicates. The first saliva sample was analyzed using the concentration step as outlined in the *Electrophoretic Conditions* section. The second saliva sample, which was not subjected to the concentration step, was analyzed by hydrodynamic introduction of the sample into the CE system (with a sample volume of approximately 2% of the total capillary volume). Representative electropherograms of human saliva obtained using the stacking method and a simple CE method are presented in Figure 10. The SEF values, calculated from the equation above, for LA, Hcy, CysGly, and Cys were 150, 32, 55, and 42, respectively. The SEF value determined for LA is significantly higher compared to Hcy, Cys, and CysGly. This can be attributed to the distinct chemical structure of LA, which contains a cyclic disulfide ring and exhibits greater hydrophobicity. Additionally, the physicochemical properties of LA likely promote more efficient on-capillary stacking under the applied electrophoretic conditions, leading to enhanced signal intensity. In contrast, the lower SEF values observed for Hcy, Cys, and CysGly are likely due to their smaller molecular size, higher polarity, which reduces their stacking efficiency.

### 2.3. Greenness Assessment of the CE-UV Method

**Figure 11:**
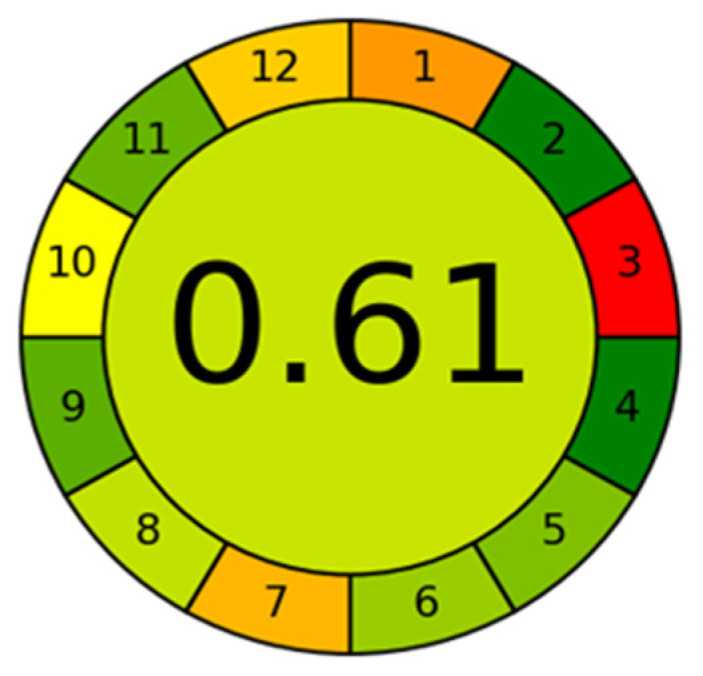
Calculated greenness of the CE-UV method.

The assessment of the greenness of analytical procedures is becoming increasingly important in the development of analytical methods. Green analytical chemistry aims to make analytical procedures more environmentally friendly and safer for people. In this study, the AGREE—Analytical GREEnness (version 0.5 beta) [15,22,25] approach and software were used to evaluate the greenness of the analytical procedures. The calculated greenness score for the presented method is 0.61, as shown in Figure 11.

Considering each of the twelve GAC principles, the CE method with UV detection for the determination of LA, Hcy, CysGly, and Cys was evaluated. Equal weights were assigned to all twelve principles. The procedure involves external sample processing with a reduced number of steps (principle 1). The volume of saliva used is 0.05 mL (principle 2). The analytical device is placed off-line (principle 3). The number of separate steps is three, including disulfide reduction with derivatization, deproteinization combined with centrifugation, and electrophoretic analysis (principle 4). The procedure is semi-autonomous and employs miniaturized sample preparation methods (principle 5). A derivatization step is required during the analysis (principle 6). The total amount of waste is 12.34 (in grams and milliliters combined) and includes the sample itself, chemicals, and plastic vessels used for sample preparation (principle 7). Four analytes are determined in a single run, and the sample throughput is four samples per hour (principle 8). The most energy-consuming technique used is CE-UV (principle 9). Some reagents can be obtained from biological sources (principle 10). The procedure requires no more than 0.02 mL of toxic solvents (principle 11), and PCA is highly flammable, highly oxidizable, and corrosive (principle 12).

**Figure 12:**
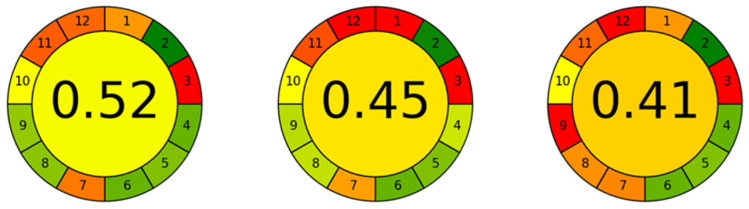
Comparison of the greenness of similar methodologies, from left to right [11,12,13].

A key question is whether our methodology is greener than other methods for determining LA, Hcy, CysGly, and Cys in human saliva. Unfortunately, the compared studies do not specify the greenness of their procedures. To address this, we estimated their greenness using a dedicated calculator and the available data from the articles. As shown in Figure 12, our method (score: 0.61) outperforms HPLC-UV (score: 0.52) [11], IP-HPLC-UV (score: 0.45) [13], and GC-MS (score: 0.41) [12] in terms of environmental impact.

Specifically, the CE-UV method is regarded as environmentally friendly due to its minimal-scale chemical analysis and reduced use of hazardous chemicals. The method also provides high-throughput potential and simplifies sample preparation. Furthermore, the CE-UV technique is known for its low energy consumption.

### 2.4. Validation of the Method

The CE-UV method was comprehensively validated to confirm its reliability in determining total LA, Hcy, CysGly, and Cys. The validation process was conducted in accordance with the latest International Council for Harmonization (ICH) harmonized guidelines for bioanalytical method validation, which specify both key parameters and acceptance criteria [26]. The validation included assessment of specificity, linearity, and limit of quantification (LOQ), as well as accuracy and precision. Additionally, potential sample matrix effects and carry-over were analyzed to ensure the reliability of the results.

#### 2.4.1. System Suitability

To evaluate the performance of the system under optimized conditions, key parameters such as the repeatability of electrophoretic migration, peak shape, and limit of detection (LOD) were analyzed. These parameters were determined based on the coefficient of variation (CV) in migration time and the asymmetry coefficient. System evaluation was conducted through repeated measurements of calibration standards at the upper LOQ limit as part of the linearity analysis. The results confirmed the high stability and precision of the system, ensuring the accuracy and repeatability of the measurements. The CV values of migration time were 0.21% for LA, 0.26% for Hcy, 0.33% for CysGly, and 0.22% for Cys, respectively, meeting the acceptance criterion (≤1%). The average asymmetry coefficient reached values of 1.131 for LA, 0.871 for Hcy, 0.94 for CysGly, and 0.839 for Cys, within the acceptance range (0.8–1.5).

#### 2.4.2. Selectivity

The selectivity of the analytical method determines its ability to identify and determine analytes in the presence of potential interfering substances in a pure biological matrix. To evaluate it, it was checked whether during the migration of the analyte in the control samples, there were no signals that could indicate interference from these substances. The analysis showed no detection of signals from the added analytes (NAC and GSH), which confirms the high selectivity of the method. Moreover, despite the presence of various functional groups (–NH_2_, –COOH) in the reaction mixtures, no formation of additional CMLT derivatives was observed. CE separation of the reaction mixtures revealed no extra signals, indicating the lack of formation of undesirable adducts.

#### 2.4.3. Linearity

The linearity of the method was assessed using external calibration. Therefore, based on available literature data [2], the concentration range for monitoring the presence of the analytes under investigation was established. For this purpose, calibration curves were prepared three times over three consecutive days, including a control sample and six points in the range of 10–100 µmol/L saliva. Each sample was prepared according to the established procedure. Linearity was verified graphically by presenting the dependence of the peak height on the analyte concentration. The analysis was performed using the least squares regression model, describing the concentration–response relationship. Additionally, the correlation coefficient (R) was assessed, which reflects the proportionality of the instrument response in the tested concentration range. Detailed data, including the linear calibration range (Figure S3), regression equation, LOD, and LOQ, are presented in Table 2. The results confirmed that the peak heights of LA, Hcy, CysGly, and Cys increased proportionally with analyte concentration, and the slopes of the regression lines remained stable, without significant deviations.

#### 2.4.4. Precision and Accuracy

The precision and accuracy of the method were assessed in terms of both intraday and interday repeatability through linearity analysis. Precision was expressed as the (CV) of repeatability, while accuracy was determined by the percentage recovery of the analyte. Calculations were performed using an established formula:

$$Accuracy\left(\%\right)=\frac{measured\;amount-endogenous\;content}{added\;amount}\times 100$$

The analysis was performed on samples prepared at three different concentration levels within the calibration range. At each level, the measurements were conducted three times in a single day to determine intraday precision and accuracy and were repeated over three consecutive days to assess interday precision and accuracy. For LA, intraday and interday precision ranged from 0.22 to 4.68% and 2.44 to 4.05%, respectively, and accuracy ranged from 95.28 to 100.52% and 89.01 to 107.17%. For Hcy, intraday precision ranged from 0.45 to 4.24%, and interday precision 0.86 to 2.38%, while precision ranged from 87.91 to 102.02% and 93.53 to 114.01%, respectively. For CysGly, intraday precision ranged from 0.41 to 1.77%, and interday precision from 0.64 to 2.71%, with precision between 89.4 and 100.28% and 89.65 and 100.75%, respectively. For Cys, intraday precision was 0.20–3.03%, and interday precision was 0.3–3.46%, while precision ranged from 94.5 to 103.18% and 96.79 to 101.74%, respectively. Detailed results are presented in Table 3. The obtained values are within the established acceptance criteria, confirming that the developed method exhibits high precision and accuracy.

#### 2.4.5. The Limit of Quantification

As part of the evaluation of the precision and accuracy of the calibration range, the analysis of the LOQ was also performed. The LOQ values were 1 µmol/L saliva for LA, 0.17 µmol/L saliva for Hcy, 0.11 µmol/L saliva for CysGly, and 0.10 µmol/L saliva for Cys. The estimated values were close to the LOQ determined by the experimental method based on the signal-to-noise ratio (S/N = 10). In contrast, LOD was determined experimentally as the concentration corresponding to an analytical signal three times higher than the baseline noise. LOD values were 0.4 µmol/L saliva for LA, 0.08 µmol/L saliva for Hcy, 0.04 µmol/L saliva for CysGly, and 0.03 µmol/L saliva for Cys. The LOQ comparison showed that the obtained results are similar to those obtained in the HPLC-UV [11] and GC-MS [12] procedures and, at the same time, lower than in the case of the IP-HPLC-UV [13] procedure. Detailed data are presented in Table 1.

#### 2.4.6. Carry-Over Effect

In analytical methods, carry-over can result in the transfer of analytes from one sample to another, potentially affecting the accuracy and precision of the determinations. In this study, the potential occurrence of this phenomenon was assessed by linearity analysis. To verify the carry-over effect, control samples of the standard solution were analyzed immediately after the calibration standards. In all cases, the signal from the control samples remained at background level, confirming the effective elimination of carry-over during method development. These results indicate that the method ensures high reliability of the determinations without the risk of cross-sample contamination.

#### 2.4.7. Matrix Effect

The matrix effect was analyzed to assess potential interference of the analyte signal caused by the presence of other, often difficult to identify, sample components. Saliva samples collected from healthy volunteers were used for the evaluation, and the analysis involved comparing calibration curves obtained from different sources with the calibration curve for the entire matrix. Additionally, the selectivity of the method and the stability of the results after sample dilution were assessed. The CVs of the slopes of the regression lines were: 14.04% for LA, 1.89% for Hcy, 6.44% for CysGly, and 5.32% for Cys, suggesting that the matrix effect had no significant impact on the analytical results. To more precisely determine analyte levels in saliva samples, the standard addition method was applied, which confirmed the reliability and precision of the measurement procedure.

#### 2.4.8. Reinjection Reproducibility

The repeatability of the method was assessed in terms of the precision and accuracy of the intraday calibration curves. The primary objective of this analysis was to verify that the prepared samples were adequately stable during storage before reinjection. The repeatability of the sample reinjection was assessed by performing at least five replicate injections of the calibration standards at different analyte concentrations. The CVs for the repeatability of the reinjection at a concentration of 3 µmol/L in saliva samples were 1.55% for LA, 1.05% for Hcy, 1.34% for CysGly, and 0.27% for Cys. At a concentration of 30 µmol/L, the CV values were: 1.63% for LA, 0.59% for Hcy, 0.76% for CysGly, and 1.08% for Cys. These results indicate a high repeatability of the method, regardless of the analyte concentration in the samples.

### 2.5. Application of the Method

**Figure 13:**
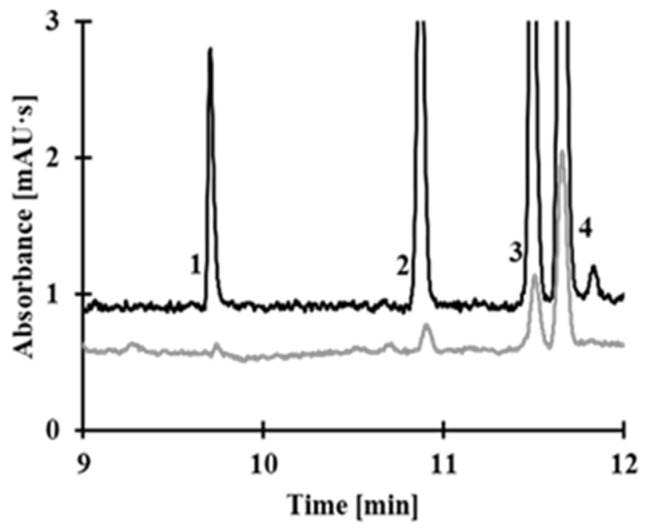
Representative electropherograms of human saliva (grey line) and human saliva spiked with 3 μmol/L of each analyte (black line). Peaks: 1 α-LA, 2 Hcy, 3 CysGly, and 4 Cys.

After validation, a CE-UV method was used to analyze saliva samples for LA, Hcy, CysGly, and Cys content. The study was conducted with a group of eleven apparently healthy volunteers from an ethnically homogeneous population. An external standard addition method was employed to determine salivary analyte levels in test samples, following the procedures described in the *Saliva Sample Preparation* and *Electrophoretic Conditions* Sections. Estimated concentrations of Hcy, CysGly, and Cys in saliva, based on triplicate analyses of specific samples from individual sources, were within the typical range for healthy subjects, ranging from 0.55 to 13.76 µmol/L, 0.89 to 9.29 µmol/L, and 1.73 to 12.99 µmol/L, respectively. No LA-derived peaks were observed in the native saliva samples, as the volunteers had not been exposed to any analytes prior to saliva sampling. Representative electropherograms for saliva and enriched saliva are shown in Figure 13.

## 3. Materials and Methods

### 3.1. Reagents and Materials

In general, chemicals used in this study were commercially available and of at least analytical reagent grade. Sodium hydroxide (NaOH), hydrochloric acid (HCl), and lithium acetate (CH_3_COOLi) were purchased from POCH (Gliwice, Poland). Sodium hydrogen phosphate heptahydrate (Na_2_HPO_4_·7H_2_O), sodium dihydrogen phosphate dihydrate (NaH_2_PO_4_·2H_2_O), acetic acid (CH_3_COOH), tris(2-carboxyethyl)phosphine hydrochloride (TCEP), trichloroacetic acid (TCA), α-lipoic acid (LA), cysteinylglycine (CysGly), and symmetrical disulfides of homocystine (Hcy)_2_ and cystine (Cys)_2_ were from Sigma-Aldrich (St. Louis, MO, USA). HPLC-grade acetonitrile (ACN) and perchloric acid (PCA) were sourced from J.T. Baker (Deventer, The Netherlands). 2-chloro-1-methyllepidinium tetrafluoroborate (CMLT) was prepared in our laboratory, as previously described [21]. The titration system was calibrated with standard pH solutions. All solutions were prepared with purified water obtained from our laboratory prior to use.

### 3.2. Instrumentation

For all CE-UV experiments, an Agilent 7100 CE System (Agilent Technologies, Waldbronn, Germany) equipped with a UV absorbance diode array detector and an automatic injector was used. Instrument control, data acquisition, and analysis were performed using ChemStation Rev. B.04.02. SP1 software. Separation was achieved using a bare fused silica capillary (Polymicro Technologies, Phoenix, AZ, USA) with a total length of 100 cm (effective length of 91.5 cm) and an inner diameter of 75 µm.

For HPLC-UV based experiments, an Agilent 1220 Infinity LC system equipped with a binary pump integrated with a two-channel vacuum degasser, an autosampler, a temperature-controlled column compartment, and a UV detector (Agilent Technologies, Waldbronn, Germany) was used. Instrument control, data acquisition, and analysis were performed using OpenLAB CDS Rev. B.04.03 SP1 software. The analytes were separated on ZORBAX SB-C18 column (150 × 4.6 mm, 5.0 μm) from Agilent Technologies (Waldbronn, Germany).

The pH of the solutions was adjusted using a FiveEasy F-20 pH meter from Mettler Toledo (Greifensee, Switzerland). Deionized water used for all experiments was purified using a Direct-Q 3 UV water purification system (Millipore, Vienna, Austria). During the study, a Mikro 220R centrifuge with a fast cool function (Hettich Zentrifugen, Tuttlingen, Germany) was used. Samples were stored in an ultra-low-temperature freezer (Panasonic Healthcare Co., Sakata, Japan).

### 3.3. Stock Solutions

All solutions were prepared with HPLC-gradient grade (in)organic solvents or ultrapure deionized water.

The stock solutions of 0.1 mol/L (Hcy)_2_, (Cys)_2_, and CysGly were prepared in 1 mol/L HCl, while stock solution of 0.1 mol/L LA was prepared in 1 mol/L NaOH. These solutions were stored at 4 °C for no longer than 7 days without any noticeable change in analyte content. The solutions were stored in polypropylene (PP) microcentrifuge tubes. Working solutions of the analytes were prepared daily by diluting the corresponding stock solution with 0.2 mol/L phosphate buffer (PB), pH 7.8, as needed, and were processed without delay.

The stock solutions of 0.25 mol/L TCEP and 0.025 mol/L CMLT were prepared by dissolving the appropriate amounts of TCEP and CMLT powders in deionized water, as needed, and processed without delay. The solution was stored in a PP tube at 4 °C for no longer than 30 min (Figure 5).

A stock solution of 0.2 mol/L PB buffer at pH 7.8 was prepared by dissolving an appropriate amount of sodium hydrogen phosphate dihydrate in water, followed by pH adjustment with an appropriate volume of 0.2 mol/L sodium dihydrogen phosphate solution using potentiometric titration. The PB buffer was prepared weekly and stored in a tightly sealed glass bottle at room temperature.

The mobile phase for the HPLC-UV experiments, consisting of 0.1 mol/L TCA at pH 1.7, was prepared by dissolving the appropriate amount of solid substance in water and adjusting the pH with sodium hydroxide (1 mol/L) using potentiometric titration. The solution was prepared weekly and stored in a tightly sealed amber glass bottle at room temperature.

The BGE of 0.5 mol/L lithium acetate buffer at pH 3.5 was prepared by dissolving an appropriate amount of lithium acetate in water, followed by pH adjustment with an appropriate volume of 0.5 mol/L acetic acid solution using potentiometric titration. The BGE was prepared weekly and stored in a tightly sealed glass bottle at room temperature.

Other solutions, including sodium hydroxide (1 mol/L), were prepared by dissolving an appropriate amount in water. PCA (3 mol/L) and hydrochloric acid (1 mol/L) were prepared by diluting standard solutions with water. All these solutions were stored in tightly sealed glass flasks at ambient temperature and used within 1 month.

### 3.4. Biological Samples Collection

First, early morning saliva samples (approximately 2 mL) were collected from individuals after overnight fasting, before brushing their teeth, and at least 15 min after rinsing their mouths with water. Unstimulated saliva samples were obtained by asking donors to expectorate into a sterile container. The samples were then cooled on ice and delivered to the laboratory within 3 h of collection. The saliva samples were clarified by centrifugation (12,000× *g*, 15 min, 4 °C), and the resulting ultrafiltrate was stored at −80 °C until analysis. Samples were processed without delay, immediately after thawing at room temperature, using the procedures described herein.

A group of eleven apparently healthy voluntary individuals (7 female and 4 male, 23–59 years old) participated in the study. The control subjects, who belonged to an ethnically homogeneous group, were not supplemented with the analytes or their precursors prior to sample collection. Additionally, no medications were allowed. All participants in the study declared that, to the best of their knowledge, none of them were suffering from any disease.

### 3.5. Saliva Sample Preparation

An amount of 50 µL of obtained salivary ultrafiltrate was mixed with 100 µL of 0.2 mol/L PB buffer (pH 7.8) and 5 µL of a mixture containing 0.25 mol/L TCEP and 0.025 mol/L CMLT. The sample was then incubated at room temperature for 5 min. After incubation, the reaction mixture was treated with 15 µL of 3 mol/L PCA and centrifuged at 12,000× *g* for 10 min at 4 °C. Finally, the resulting supernatant was subjected to electrophoretic or chromatographic analysis under the conditions described in the *Electrophoretic Conditions* and *Chromatographic Conditions* Sections.

### 3.6. Chromatographic Conditions

Saliva samples, prepared for optimizing the sample preparation conditions, were analyzed using a previously published HPLC-UV assay by Piechocka et al. [18]. Chromatographic separations were performed at room temperature using linear gradient elution (0–8 min, 11–40% B; 8–12 min, 40–11% B; 12–14 min, 11% B), with the mobile phase consisted of 0.1 mol/L TCA (pH 1.7, solvent A) and ACN (solvent B), delivered at the flow rate of 1 mL/min. The effluent was monitored with a UV detector at 355 nm (bandwidth 4 nm) using 390 nm (bandwidth 20 nm) as a reference wavelength.

### 3.7. Electrophoretic Conditions

In case of a new capillary, the preconditioning procedure involved flushing with 1 mol/L NaOH solution for 20 min, followed by 20 min with 0.1 mol/L NaOH solution, 2 min with deionized water, and finally, 30 min with BGE. Every next day of work, the capillary was flushed for 5 min with 1 mol/L NaOH solution, followed by 20 min with 0.1 mol/L NaOH solution, 2 min with deionized water, and 30 min with BGE. At the end of each day, the capillary was flushed for 20 min with deionized water, and the capillary ends were left in water overnight.

During electrophoretic analysis, a 0.5 mol/L lithium acetate buffer at pH 3.5 was used as the BGE. Separation was performed under positive polarity at 30 kV (~30 µA) and 25 °C. After conditioning the capillary with the BGE, a 100 s electrokinetic injection of the final analytical solution was carried out at 20 kV, followed by a 100 s injection of 0.6 mol/L HCl at 20 kV. UV detection was performed at 355 nm with a 5 nm bandwidth, using 450 nm with a 60 nm bandwidth as the reference wavelength for all analytes.

## 4. Conclusions

A new, fast, and simple method for the simultaneous determination of total LA, Hcy, CysGly, and Cys in human saliva using CE with UV detection and pH-mediated sample stacking was developed. This method features a simplified sample preparation procedure, including disulfide bond reduction, derivatization, deproteinization, and CE-UV analysis. A notable advantage is the ability to simultaneously reduce disulfide bonds and perform derivatization. Another advantage is minimization of the consumption of both reagents and solvents, resulting in lower analysis costs and less waste. Thus, the method is consistent with the principles of green chemistry. Additionally, the stability and durability of the reducing-derivatizing mixture were demonstrated, allowing for the preparation of multiple samples in a single analytical run. The procedure is straightforward and does not require complex equipment, while maintaining a wide linearity range, high sensitivity, precision, and accuracy. The method is characterized by high selectivity and resolution similar to HPLC methods. Therefore, it represents a very good and environmentally friendly alternative to HPLC. The main advantages of this method are simple preparation of the sample and short processing time (~13 min), making it more convenient than previously developed HPLC-based methods, which require over 21 min (Table 1). Moreover, the reduced number of preparation steps shortens sample preparation time to just 15 min, the shortest reported so far. The total analysis time, including CE separation, is approximately 28 min. These features make the method well-suited for routine analysis of saliva for LA, Hcy, CysGly, and Cys content. In summary, we believe that the developed method is fit for purpose, and due to its lower reagent consumption and reduced waste generation, represents an effective and ecological alternative to classical HPLC methods.

## Figures and Tables

**Table 1:** The comparison of selected methods for the determination of LA, Hcy, CysGly, and Cys in human saliva.

Analyte	Method	Volume Sample * [µL]	Chemicals Consumption [µL]	Linearity [µmol/L]	LOQ [µmol/L]	Sample Preparation Time [min]	Overall Analysis Time ** [min]	Method Reference
LA	GC-MS	50	320	n.a.	n.a.	35	65	[12]
Hcy				0.5–20	0.1			
CysGly				n.a.	n.a.			
Cys				0.5–20	0.1			
LA	HPLC-UV	50	120	n.a.	n.a.	23	31	[11]
Hcy				0.1–20	0.05			
CysGly				0.25–50	0.12			
Cys				5–300	0.08			
LA	IP-HPLC	100	300	n.a.	n.a.	40	51	[13]
Hcy				0.15–500	0.15			
CysGly				0.15–500	0.15			
Cys				0.15–500	0.12			
LA	CE-UV	50	170	3–30	1	15	28	Present method
Hcy				3–30	0.17			
CysGly				3–30	0.11			
Cys				3–30	0.1			

n.a.—no data available; * the volume of chemicals used per sample referring to sample preparation step; ** overall analysis time corresponding to sample preparation and chromatographic/electrophoretic separation time.

**Table 2:** Validation data corresponding to intra-assay measurements (*n* = 3). LOD, limit of detection; LOQ, limit of quantification; R, correlation coefficient.

Analyte	Equations of the Calibration Curve	Calibration Concentration Range [µmol/L]	R	LOD [µmol/L]	LOQ [µmol/L]
LA	y = (1.0172 ± 0.1356)x + 0.7578 ± 0.0747	3–30	0.9992	0.4	1
Hcy	y = (3.3225 ± 0.0886)x + 0.3791 ± 0.1978	3–30	0.9994	0.08	0.17
CysGly	y = (3.9626 ± 0.0528)x + 1.121 ± 0.03684	3–30	0.9997	0.04	0.11
Cys	y = (5.4346 ± 0.0543)x + 1.2468 ± 0.07495	3–30	0.9998	0.03	0.10

**Table 3:** Validation data—results.

Analyte	Added * (µmol/L)	Intraday			Interday		
		Found ± SD (µmol/L)	CV (%)	Accuracy (%)	Found ± SD (µmol/L)	CV (%)	Accuracy (%)
LA	3	2.86 ± 0.13	4.68	95.28	2.67 ± 0.11	4.05	89.01
	12	12.06 ± 0.38	3.14	100.52	10.69 ± 0.37	3.43	89.05
	18	17.98 ± 0.04	0.22	99.89	19.29 ± 0.47	2.44	107.17
	30	30.47 ± 0.42	1.38	101.57	29.90 ± 0.78	2.61	99.66
Hcy	3	2.99 ± 0.06	2.04	87.91	3.42 ± 0.08	2.38	114.01
	12	11.42 ± 0.48	4.24	95.15	13.41 ± 0.12	0.87	111.78
	18	18.51 ± 0.08	0.45	102.02	16.84 ± 0.14	0.86	93.53
	30	30.05 ± 0.54	1.78	99.84	30.52 ± 0.19	2.71	101.75
CysGly	3	2.93 ± 0.04	1.37	89.40	2.68 ± 0.06	2.18	89.65
	12	11.73 ± 0.21	1.77	97.70	12.09 ± 0.18	1.45	100.75
	18	17.89 ± 0.07	0.41	98.70	18.05 ± 0.25	1.40	100.27
	30	30.29 ± 0.37	1.24	100.28	29.38 ± 0.19	0.64	97.95
Cys	3	3.16 ± 0.07	2.36	94.50	2.9 ± 0.02	0.79	96.79
	12	11.79 ± 0.36	3.03	98.24	12.21 ± 0.14	1.11	101.74
	18	18.57 ± 0.06	0.20	103.18	17.64 ± 0.05	0.30	97.98
	30	29.81 ± 0.42	0.43	98.15	29.73 ± 1.03	3.46	99.11

* *n* = 3.

## Data Availability

Essential data are contained within the article and Supplementary Materials. Additionally, the dataset generated and analyzed during this study, which contributed to the article, is available from the corresponding authors upon reasonable request, provided that such requests do not compromise intellectual property interests. Saliva samples are not available from the authors.

## Supplementary Materials

The following supporting information can be downloaded at: https://www.mdpi.com/article/10.3390/molecules30153129/s1. Figure S1. Stability of 2-*S*-lepidinium derivatives of Hcy, Cys, CysGly, and LA at (a) room temperature, (b) 4 °C, (c) −20 °C, and (d) −80 °C as a function of time; expressed as a peak area of each 2-*S*-lepidinium derivative. Error bars represent the standard deviation of the data (*n* = 3). Figure S2. Freeze–thaw stability of 2-*S*-lepidinium derivatives of Hcy, Cys, CysGly, and LA; expressed as a peak area of each 2-*S*-lepidinium derivative. Error bars represent the standard deviation of the data (*n* = 3). Figure S3. Calibration curves.

## Abbreviations

ACN	Acetonitrile
BGE	Background electrolyte
CE	Capillary electrophoresis
CMLT	2-chloro-1-methyllepidinium tetrafluoroborate
CV	Coefficient of variation
Cys	Cysteine
Cys_2_	Cystine
CysGly	Cysteinylglycine
GAC	Green Analytical Chemistry
GC	Gas chromatography
GSH	Glutathione
Hcy	Homocysteine
Hcy_2_	Homocystine
HPLC	High-performance liquid chromatography
ICH	International Council for Harmonization
IP-HPLC	Ion-pairing high-performance liquid chromatography
LA	α-lipoic acid
LOD	Limit of determination
LOQ	Limit of quantification
LIF	Laser-induced fluorescence
MS	Mass spectrometry
NAC	N-acetyl-cysteine
PB	Phosphate buffer
PCA	Perchloric acid
PP	Polypropylene
R	Correlation coefficient
SEF	Sensitivity enhancement factory
TCA	Trichloroacetic acid
TCEP	Tris(2-carboxyethyl)phosphine
UV	Ultraviolet detection
γ-Glu-Cys	γ-glutamyl-cysteine

## References

[1] Gallardo E., Queiroz J.A. (2008). The Role of Alternative Specimens in Toxicological Analysis. Biomed. Chromatogrpahy.

[2] Piechocka J., Wrońska M., Głowacki R. (2020). Chromatographic Strategies for the Determination of Aminothiols in Human Saliva. Trends Anal. Chem..

[3] Oliveira P.V., Laurindo F.R.M. (2018). Implications of Plasma Thiol Redox in Disease. Clin. Sci..

[4] Elmongy H., Abdel-Rehim M. (2016). Saliva as an Alternative Specimen to Plasma for Drug Bioanalysis: A Review. Trends Anal. Chem..

[5] De Almeida P.D.V., Grégio A.M.T., Machado M.Â.N., De Lima A.A.S., Azevedo L.R. (2008). Saliva Composition and Functions: A Comprehensive Review. J. Contemp. Dent. Pract..

[6] Carlucci F., Tabucchi A. (2009). Capillary Electrophoresis in the Evaluation of Aminothiols in Body Fluids. J. Chromatogr. B Anal. Technol. Biomed. Life Sci..

[7] Ivanov A.V., Popov M.A., Aleksandrin V.V., Pudova P.A., Galdobina M.P., Metelkin A.A., Kruglova M.P., Maslennikov R.A., Silina E.V., Stupin V.A. (2023). Simultaneous Determination of Cystine and Other Free Aminothiols in Blood Plasma Using Capillary Electrophoresis with pH-Mediated Stacking. Electrophoresis.

[8] Zinellu A., Sotgia S., Scanu B., Pisanu E., Sanna M., Sati S., Deiana L., Sengupta S., Carru C. (2010). Determination of Homocysteine Thiolactone, Reduced Homocysteine, Homocystine, Homocysteine-Cysteine Mixed Disulfide, Cysteine and Cystine in a Reaction Mixture by Overimposed Pressure/Voltage Capillary Electrophoresis. Talanta.

[9] Kubalczyk P., Bald E., Furmaniak P., Głowacki R. (2014). Simultaneous Determination of Total Homocysteine and Cysteine in Human Plasma by Capillary Zone Electrophoresis with pH-Mediated Sample Stacking. Anal. Methods.

[10] Kubalczyk P., Głowacki R. (2017). Determination of Lipoic Acid in Human Urine by Capillary Zone Electrophoresis. Electrophoresis.

[11] Stachniuk J., Kubalczyk P., Furmaniak P., Głowacki R. (2016). A Versatile Method for Analysis of Saliva, Plasma and Urine for Total Thiols Using HPLC with UV Detection. Talanta.

[12] Piechocka J., Wieczorek M., Głowacki R. (2020). Gas Chromatography–Mass Spectrometry Based Approach for the Determination of Methionine-Related Sulfur-Containing Compounds in Human Saliva. Int. J. Mol. Sci..

[13] Zhang W., Li P., Geng Q., Duan Y., Guo M., Cao Y. (2014). Simultaneous Determination of Glutathione, Cysteine, Homocysteine, and Cysteinylglycine in Biological Fluids by Ion-Pairing High-Performance Liquid Chromatography Coupled with Precolumn Derivatization. J. Agric. Food Chem..

[14] Hodáková J., Preisler J., Foret F., Kubáň P. (2015). Sensitive Determination of Glutathione in Biological Samples by Capillary Electrophoresis with Green (515nm) Laser-Induced Fluorescence Detection. J. Chromatogr. A.

[15] Pena-Pereira F., Wojnowski W., Tobiszewski M. (2020). AGREE—Analytical GREEnness Metric Approach and Software. Anal. Chem..

[16] Isokawa M., Kanamori T., Funatsu T., Tsunoda M. (2014). Analytical Methods Involving Separation Techniques for Determination of Low-Molecular-Weight Biothiols in Human Plasma and Blood. J. Chromatogr. B.

[17] Głowacki R., Piechocka J., Bald E., Chwatko G., Buszewski B., Baranowska I. (2022). Application of Separation Techniques in Analytics of Biologically Relevant Sulfur Compounds. Handbook of Bioanalytics.

[18] Piechocka J., Wyszczelska-Rokiel M., Głowacki R. (2023). Simultaneous Determination of 2-(3-Hydroxy-5-Phosphonooxymethyl-2-Methyl-4-Pyridyl)-1,3-Thiazolidine-4-Carboxylic Acid and Main Plasma Aminothiols by HPLC–UV Based Method. Sci. Rep..

[19] Roblegg E., Coughran A., Sirjani D. (2019). Saliva: An All-Rounder of Our Body. Eur. J. Pharm. Biopharm..

[20] Furmaniak P., Kubalczyk P., Stachniuk J., Głowacki R. (2016). Novel MEKC Method for Determination of Sodium 2-Mercaptoethanesulfonate in Human Plasma with in-Capillary Derivatization and UV Detection. J. Chromatogr. B.

[21] Głowacki R., Borowczyk K., Bald E. (2014). Determination of Nε-Homocysteinyl-Lysine and γ-Glutamylcysteine in Plasma by Liquid Chromatography with UV-Detection. J. Anal. Chem..

[22] Wojnowski W., Tobiszewski M., Pena-Pereira F., Psillakis E. (2022). AGREEprep—Analytical Greenness Metric for Sample Preparation. TrAC Trends Anal. Chem..

[23] Kubalczyk P., Bald E., Buszewski B., Dziubakiewicz E., Szumski M. (2013). Methods of Analyte Concentration in a Capillary. Electromigration Techniques Theory and Practice.

[24] Hadwiger M.E., Torchia S.R., Park S., Biggin M.E., Lunte C.E. (1996). Optimization of the Separation and Detection of the Enantiomers of Isoproterenol in Microdialysis Samples by Cyclodextrin-Modified Capillary Electrophoresis Using Electrochemical Detection. J. Chromatogr. B Biomed. Appl..

[25] Pena-Pereira F., Tobiszewski M., Wojnowski W., Psillakis E. (2022). A Tutorial on AGREEprep an Analytical Greenness Metric for Sample Preparation. Adv. Sample Prep..

[26] ICH (2022). M10 Guideline for Bioanalytical Method Validation and Study Sample Analysis.

