# Supplementary material for: Fast, Simple and Accurate Method for Simultaneous Determination of α-Lipoic Acid and Selected Thiols in Human Saliva by Capillary Electrophoresis with UV Detection and pH-Mediated Sample Stacking
Source: Molecules. 2025 Jul 25;30(15):3129. doi: 10.3390/molecules30153129 (PMC12348659; doi:10.3390/molecules30153129)
Supplement: Supplementary file 1 [file molecules-30-03129-s001.zip › molecules-3721830-supplementary.pdf]

molecules

MDPI

Article

Fast, Simple and Accurate Method for Simultaneous Determination of A-Lipoic Acid and Selected Thiols in Human Saliva by Capillary Electrophoresis with Uv Detection and Ph-Mediated Sample Stacking

Urszula Sudomir[1,2], Justyna Piechocka[1]*, Rafał Głowacki[1], Paweł Kubalczyk[1]*

[1] University of Lodz, Faculty of Chemistry, Department of Environmental Chemistry, 163/165 Pomorska Str., 90-236 Łódź, Poland; urszula.sudomir@edu.uni.lodz.pl (U.S.), pawel.kubalczyk@chemia.uni.lodz.pl (P.K.), justyna.piechocka@chemia.uni.lodz.pl (J.P.), rafal.glowacki@chemia.uni.lodz.pl (R.G.)

[2] University of Lodz, Doctoral School of Exact and Natural Sciences, 12/16 Banacha Str., 90-237 Łódź, Poland;

* Correspondence: pawel.kubalczyk@chemia.uni.lodz.pl (P.K.), justyna.piechocka@chemia.uni.lodz.pl (J.P.); Tel.: +48-42-635 5844; +48-42-635 5790, Fax: +48-42-635 5832

Supplementary Materials: The following supporting information can be downloaded at: https://www.mdpi.com/article/doi/s1 **Figure S1.** Stability of 2-*S*-lepidinium derivatives of Hcy, Cys, CysGly, and LA at (a) room temperature, (b) 4 °C, (c) -20 °C, and (d) -80 °C as a function of time; expressed as a peak area of each 2-*S*-lepidinium derivative. Error bars represent the standard deviation of the data (n=3). **Figure S2.** Freeze-thaw stability of 2-*S*-lepidinium derivatives of Hcy, Cys, CysGly, and LA; expressed as a peak area of each 2-*S*-lepidinium derivative. Error bars represent the standard deviation of the data (n=3). **Figure S3.** Calibration curves.

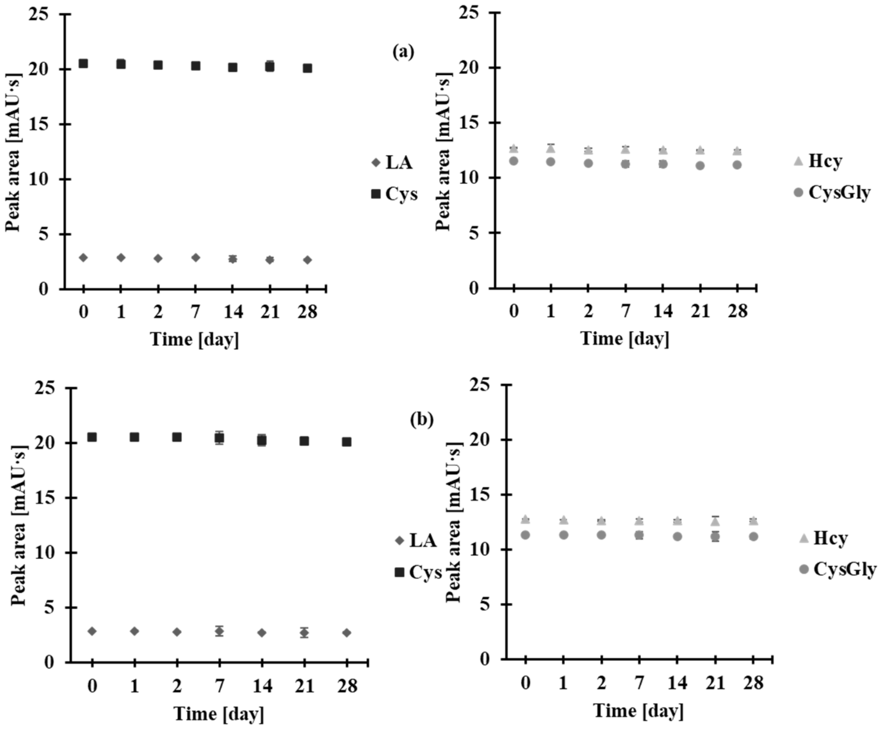

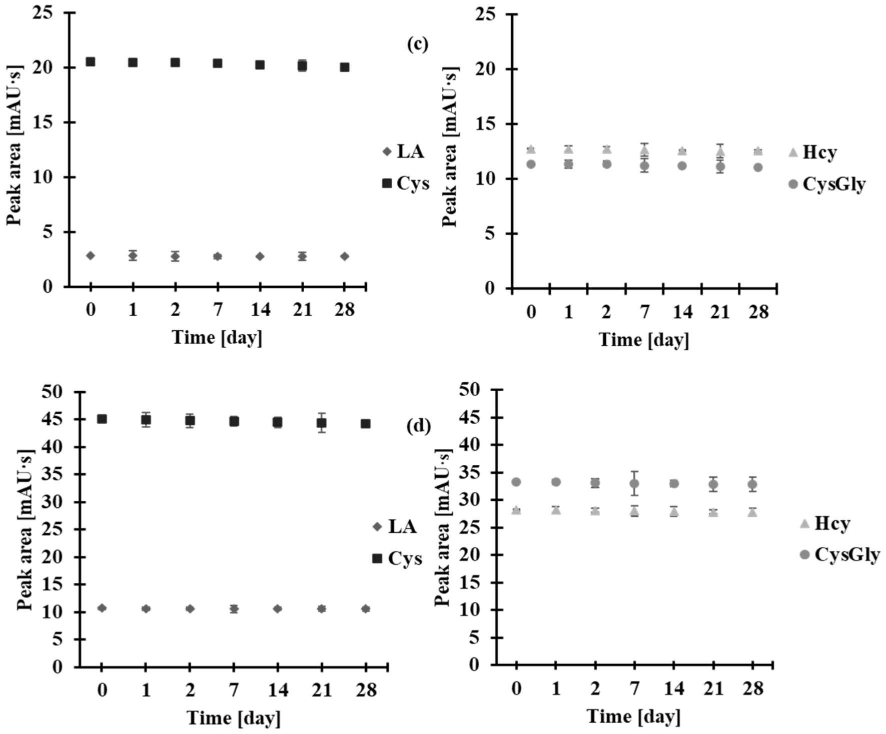

Figure S1. Stability of 2-*S*-lepidinium derivatives of Hcy, Cys, CysGly, and LA at (a) room temperature, (b) 4 °C, (c) -20 °C, and (d) -80 °C as a function of time; expressed as a peak area of each 2-*S*-lepidinium derivative. Error bars represent the standard deviation of the data (n=3).

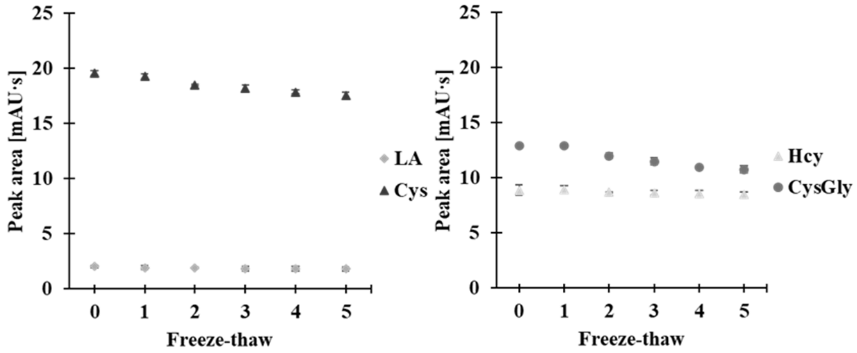

Figure S2. Freeze-thaw stability of 2-*S*-lepidinium derivatives of Hcy, Cys, CysGly, and LA; expressed as a peak area of each 2-*S*-lepidinium derivative. Error bars represent the standard deviation of the data (n=3).

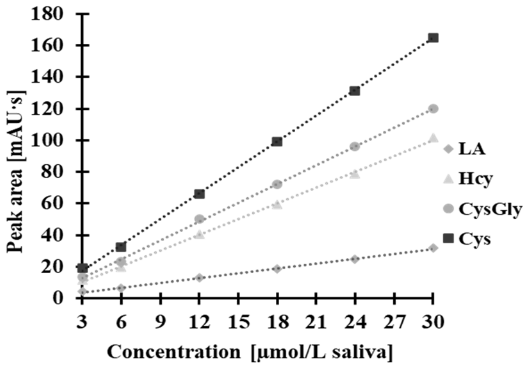

Figure S3. Calibration curves.
